# Renal Cell Carcinoma in Pregnancy With Breast Metastasis: A Case Report and Literature Review

**DOI:** 10.7759/cureus.92550

**Published:** 2025-09-17

**Authors:** Ferit Aslan, Burcu Erkılıç, Gülenay Türkmen, Elif Günaydın, Ceyhun Hasanov

**Affiliations:** 1 Medical Oncology, Medical Park Batikent Hospital, Ankara, TUR; 2 Internal Medicine, Yüksek İhtisas University, Ankara, TUR; 3 Obstetrics and Gynecology, Medical Park Batikent Hospital, Ankara, TUR; 4 Department of Radiology, Yüksek İhtisas University, Ankara, TUR; 5 Radiology, Medical Park Batikent Hospital, Ankara, TUR; 6 Urology, Ankara Magnet Hospital, Ankara, TUR

**Keywords:** breast metastasis, clear cell carcinoma, fbxw7 mutation, nf1 mutation, pregnancy, renal cell carcinoma, systemic therapy

## Abstract

Renal cell carcinoma (RCC) during pregnancy is exceedingly rare and presents significant diagnostic and therapeutic challenges. We report the case of a 45-year-old gravida 3 woman in whom a right renal mass was incidentally identified during the second trimester. At 34 weeks of gestation, she underwent cesarean delivery followed by right radical nephrectomy. Histopathology revealed a 12 cm clear cell RCC with renal sinus invasion. Postpartum staging demonstrated multiple pulmonary metastases, and systemic therapy was initiated with cabozantinib plus nivolumab. Despite temporary stabilization, disease progression occurred, and subsequent lines of therapy included axitinib, everolimus, and sunitinib. During follow-up, a newly developed breast mass was confirmed as metastatic RCC, representing an exceptionally rare metastatic site. Comprehensive molecular profiling revealed pathogenic variants in NF1 and FBXW7, alterations that are associated with aggressive tumor biology and resistance to systemic therapy. Despite multidisciplinary management, the patient experienced rapid disease progression and died 13 months after diagnosis. This case underscores several important clinical considerations: the difficulty of establishing a timely diagnosis of RCC in pregnancy due to overlapping maternal symptoms, the need to balance maternal and fetal safety when planning surgical intervention, and the limitations of systemic therapy in the peripartum period. Furthermore, the presence of aggressive molecular alterations may contribute to resistance and poor outcomes. The rare occurrence of breast metastasis highlights the potential for unusual dissemination patterns in RCC. Reporting such uncommon cases contributes to improved understanding of disease behavior, helps guide clinical decision-making, and emphasizes the importance of individualized, multidisciplinary management in this challenging setting.

## Introduction

Renal cell carcinoma (RCC) accounts for approximately 3% of adult malignancies and represents the most common primary renal tumor [1]. Its occurrence during pregnancy is extremely rare, affecting fewer than 0.01% of pregnancies [2]. Diagnosis is often delayed because typical symptoms such as flank pain, hematuria, or hypertension may overlap with the physiological changes of gestation [2]. The rising maternal age at conception may also contribute to an increased incidence of RCC diagnosed during pregnancy [3]. The potential influence of pregnancy-related hormones on the development of RCC remains controversial. While some studies suggest that elevated estrogen levels may stimulate renal cell proliferation, increased parity has also been associated with higher RCC risk [4]. However, definitive evidence is lacking, and aggressive tumor biology may play a more decisive role in rapid disease progression [1].

In this report, we present the case of a patient diagnosed with RCC during pregnancy who developed rapid metastatic progression, including the exceptionally rare occurrence of breast metastasis. We also review the literature regarding diagnostic and therapeutic considerations in this challenging setting.

## Case presentation

A 45-year-old gravida 3, para 3 woman with a history of hypertension controlled with methyldopa was incidentally found to have a right renal mass at 25 weeks of gestation during routine obstetric ultrasonography. Given concerns for both maternal and fetal safety, the biopsy was deferred, and management was discussed at a multidisciplinary tumor board.

At 34 weeks of gestation, the patient underwent cesarean delivery immediately followed by right radical nephrectomy. Gross examination revealed a 12 cm renal mass, and histopathology confirmed clear cell RCC (ISUP grade II) with renal sinus invasion but without perirenal fat or adrenal involvement. Immunohistochemical analysis demonstrated positivity for keratin 19, pancytokeratin, RCC marker, PAX8, and vimentin, with a proliferative index (Ki-67) of approximately 5%.

**Figure 1:**
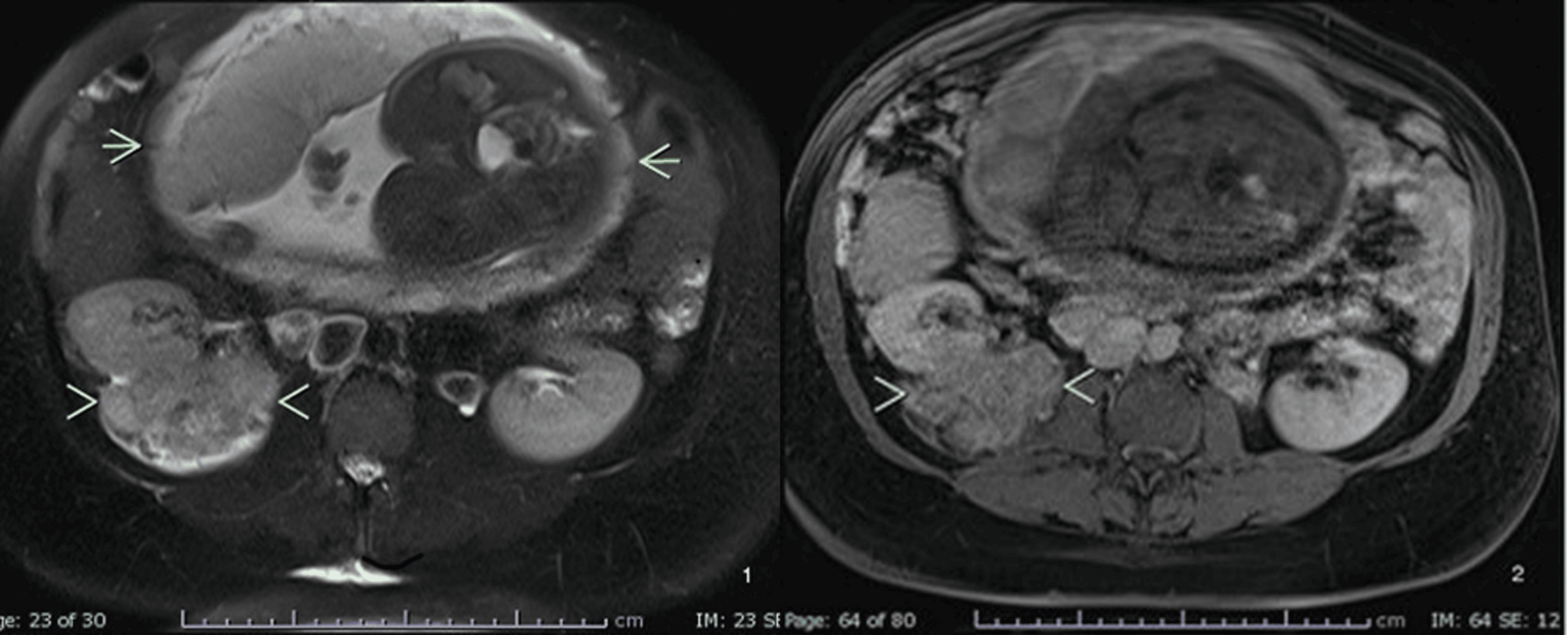
MRI at the third trimester of pregnancy revealed a large heterogeneous right-sided renal mass. On T1- and T2-weighted, MRI images, the mass is marked with arrowheads and the fetus is marked with short arrows.

During pregnancy, staging investigations were limited to ultrasonography and magnetic resonance imaging (MRI) without gadolinium. MRI performed in the third trimester demonstrated a large heterogeneous right renal mass, with the fetus clearly visible in the field of view (Figure 1).

**Figure 2:**
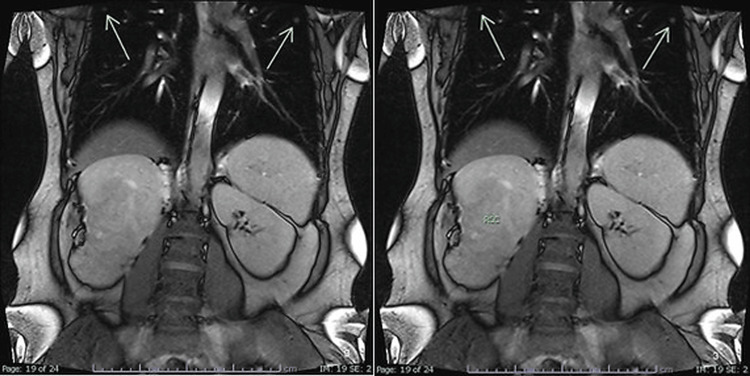
Coronal MRI images demonstrating bilateral pulmonary metastases (arrows).

**Figure 3:**
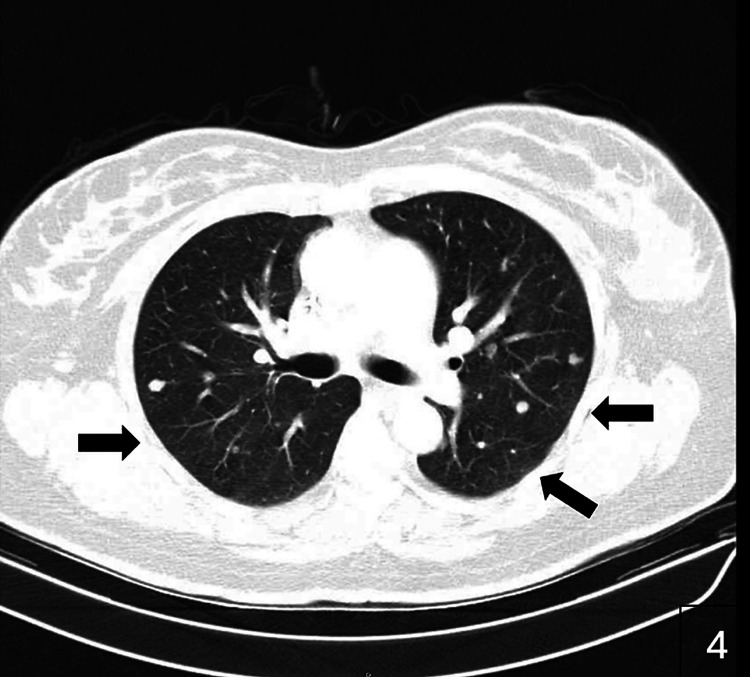
Axial CT scan showing multiple round nodules of varying sizes in both lungs, consistent with pulmonary metastases (arrows).

**Figure 4:**
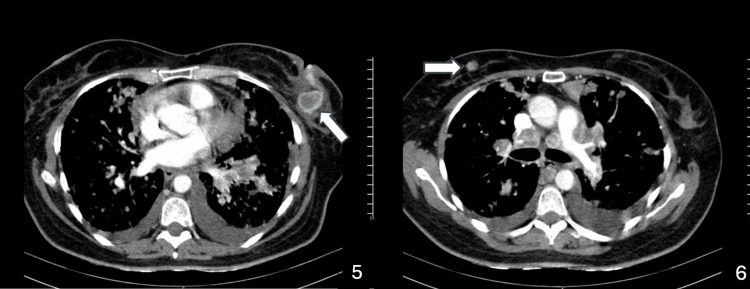
Axial CT scans demonstrating the metastatic spread of renal cell carcinoma to both breasts (arrows), with associated mediastinal lymphadenopathy and bilateral pleural effusions.

Sixteen days postpartum, an 18F-FDG PET/CT scan demonstrated multiple bilateral pulmonary nodules with FDG uptake consistent with metastatic disease. During pregnancy, the MRI performed in the third trimester demonstrated a large heterogeneous right renal mass, with the fetus visible in the field of view (Figures 1, 2). In addition, pulmonary nodules suspicious for metastatic disease were already evident in the lung sections, and postpartum imaging confirmed rapid progression with multiple bilateral lesions (Figures 3, 4). The patient was classified as IMDC intermediate risk with an ECOG performance status of 1.

Comprehensive molecular profiling revealed a low tumor mutational burden (2 muts/Mb), positive PD-L1 expression (CPS 6), and microsatellite stability. Pathogenic variants in FBXW7 (p.Glu117del) and NF1 (p.Leu1628*) were identified, along with several variants of uncertain significance, including alterations in POLE, EPHA5, NFE2L2, BRCA2, FAT4, KEAP1, FGFR1, TP53BP1, MDC1, and DDR1.

First-line systemic therapy with cabozantinib plus nivolumab was initiated postpartum, achieving disease stabilization with a progression-free survival of eight months. Upon progression with new pulmonary and mediastinal lesions, second-line axitinib was introduced but was poorly tolerated due to grade 3-4 diarrhea and provided limited benefit (progression-free survival of three months). During this period, a palpable breast mass was detected, and core needle biopsy confirmed metastatic RCC. Histopathology of bilateral breast biopsies demonstrated malignant epithelial infiltration consistent with renal origin, supported by immunohistochemistry. Imaging confirmed metastatic involvement of both breasts, accompanied by mediastinal lymphadenopathy and pleural effusion (Figures 3, 4).

**Figure 5:**
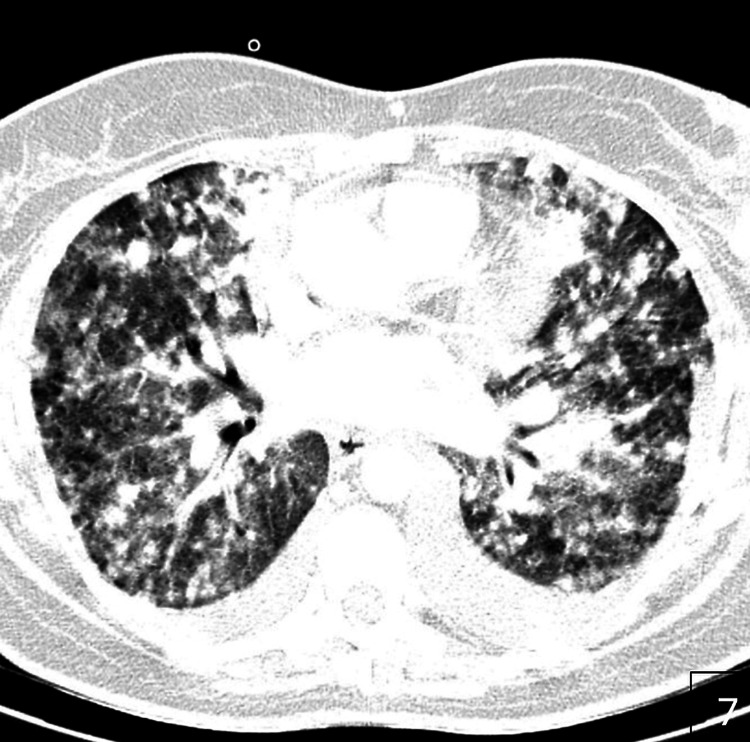
Axial CT scan demonstrating massive pulmonary metastases from renal cell carcinoma.

Third-line therapy with everolimus provided transient disease control for approximately one month, after which sunitinib was introduced. Despite treatment, the patient’s clinical condition deteriorated, and she died 13 months after the initial diagnosis. Follow-up imaging documented massive pulmonary metastases (Figure 5).

## Discussion

The management and staging of RCC during pregnancy generally follow the same AJCC/UICC TNM classification applied in non-pregnant patients, but diagnostic pathways are modified to minimize fetal risk. Ultrasound is the preferred first-line imaging modality, and MRI without gadolinium provides additional anatomical detail when necessary [5,6]. Computed tomography is usually avoided because of radiation exposure but may be considered in life-threatening situations. These limitations often result in incomplete staging during pregnancy, with comprehensive oncologic assessment deferred until the postpartum period. Epidemiological data suggest that RCC in pregnancy tends to occur in younger women, with a median age of 32-36 years [3].

Surgery remains the cornerstone of curative therapy. The second trimester is typically the safest time for non-obstetric surgery, although nephrectomy can also be performed at the time of cesarean delivery in late pregnancy [2,7]. In metastatic disease, systemic therapy with tyrosine kinase inhibitors (TKIs) and immune checkpoint inhibitors (ICIs) has improved survival in non-pregnant patients, but these drugs are contraindicated in pregnancy and during lactation due to embryo-fetal toxicity [8,9,10]. Therefore, systemic treatment is generally delayed until after delivery. In the present case, surgery was performed concomitantly with cesarean section, but aggressive disease progression was observed shortly after.

Pulmonary metastases are common in advanced RCC, and their presence frequently indicates aggressive tumor biology. In our patient, rapid progression was documented shortly after delivery, highlighting the particularly unfavorable course. Importantly, although our case was metastatic, rapid disease progression can also be observed in non-metastatic RCC with unfavorable molecular alterations, further underlining the need for vigilant follow-up

Cytoreductive nephrectomy has been evaluated in randomized trials. The CARMENA and SURTIME studies indicated that nephrectomy may benefit selected patients with low metastatic burden and favorable or intermediate risk profiles [11,12]. However, in our patient, molecular analysis revealed truncating NF1 and pathogenic FBXW7 alterations. Loss of FBXW7 function has been associated with chemotherapy resistance, increased tumor invasion, genetic instability, and poor prognosis in several malignancies, including RCC [13,14]. Similarly, NF1 truncating mutations have been correlated with aggressive tumor phenotypes and worse clinical outcomes [15]. These findings likely explain the rapid progression observed, with overall survival limited to 13 months despite multi-line systemic therapy.

Breast metastases from extramammary tumors are rare, accounting for only 0.5-2% of breast malignancies, with RCC being an exceptionally uncommon source [16]. To date, only a few dozen cases have been reported. Breast metastases usually present as circumscribed, mobile, painless masses and may mimic primary breast cancer or benign tumors. Diagnosis requires biopsy with immunohistochemistry, which in our case confirmed the renal origin. Although surgical resection may be considered for isolated lesions, prognosis is dictated by systemic disease progression.

A limitation of this case report is the absence of histopathological slide images. Unfortunately, pathology slides or digital histology photographs could not be retrieved due to institutional restrictions and the lack of archived material. Nevertheless, detailed histopathological descriptions and immunohistochemical findings have been included in the manuscript to substantiate the diagnosis.

## Conclusions

RCC during pregnancy is an extremely rare clinical entity that poses unique diagnostic and therapeutic challenges. Although surgical management remains the mainstay of treatment, systemic therapies are generally delayed until after delivery due to safety concerns. In the present case, despite timely surgery and multidisciplinary care, aggressive disease biology associated with NF1 and FBXW7 alterations led to rapid progression and poor outcomes. The occurrence of breast metastasis further underscores the potential for unusual dissemination patterns in RCC. Reporting such rare cases contributes to the literature, improves awareness, and may aid in guiding future clinical decision-making.
